# Active porous transition towards spatiotemporal control of molecular flow in a crystal membrane

**DOI:** 10.1038/ncomms9934

**Published:** 2015-11-16

**Authors:** Yuichi Takasaki, Satoshi Takamizawa

**Affiliations:** 1 Graduate School of Nanobioscience, Yokohama City University, 22-2 Seto, Kanazawa-ku, Yokohama, Kanagawa 236-0027, Japan

## Abstract

Fluidic control is an essential technology widely found in processes such as flood control in land irrigation and cell metabolism in biological tissues. In any fluidic control system, valve function is the key mechanism used to actively regulate flow and miniaturization of fluidic regulation with precise workability will be particularly vital in the development of microfluidic control. The concept of crystal engineering is alternative to processing technology in microstructure construction, as the ultimate microfluidic devices must provide molecular level control. Consequently, microporous crystals can instantly be converted to microfluidic devices if introduced in an active transformability of porous structure and geometry. Here we show that the introduction of a stress-induced martensitic transition mechanism converts a microporous molecular crystal into an active fluidic device with spatiotemporal molecular flow controllability through mechanical reorientation of subnanometre channels.

## Abstract (web summary)

Valve functionality is an essential tool for flow regulation in microfluidic systems. Here, the authors employ porous crystals which undergo mechanical reorientation of their internal subnanometer channels in order to manipulate spatiotemporal flow.

## Graphical abstract

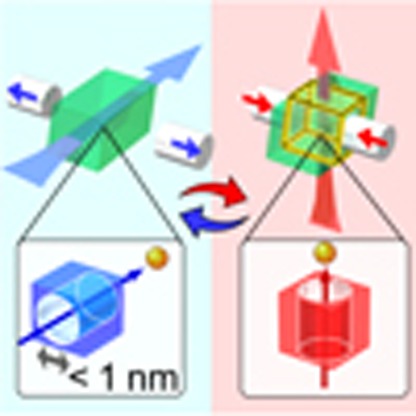

Orderly fluid transportation can be seen in a wide range of processes, from irrigation to biological tissues such as flood control or cell metabolism, through programmed pathways with adequate motive force to generate flow. Although the scale and means used in fluidic control depend on purpose, the valve function that controls the directivity and mass rate of flow is a key mechanism to actively regulate flow in any fluidic transportation operation. In microfluidic control systems^1^,^2^, precision in the ‘valve' mechanism driven by an active procedure such as magnetic^3^, electrostatic^4^ or piezoelectric^5^ actuation is especially important. Currently, gas fluidic research of man-made microfluidic-oriented devices are on a submillimetre scale^6^,^7^,^8^ and that of passages are on submicrometre scale by present-day progressing technology^9^. Although processing technologies will continue to advance, the technological limits will be continuously addressed as sizes and architectural styles change and novel methods will be explored in future micro- or nanofluidic devices. Considering the ultimate miniaturization of microfluidic systems, passage widths will reach the sizes of the fluid components and the fluid should behave as a diffusion of the included component particles. Thus, towards the theoretically finest flow control, microporous solids seem to have the fundamental requirements for such the ultimately miniature devices. They could immediately convert into attractive ‘nanofluidic' devices if some sort of molecular flow controllability is embedded in them. Therefore, we have focused on microporous molecular crystals with flexible subnanometre channels and have reported on the anisotropic gas permeation through channels^10^,^11^. In the past, the several interchangeabilities of a channel geometry by gas adsorption-induced phase transitions have been reported^12^,^13^,^14^. The remaining requirement was how to instill active controllability into such microporous crystals. We recently found a superelastic property in organic molecular crystals (organosuperelasticity)^15^,^16^ that can actively rearrange the porous structure by mechanically induced transition if the organosuperelastic crystal was porous. Herein, we report a microporous crystal with superelasticity that actively controls accurate molecular flow by means of channel rearrangement. Active transformability in a porous crystal will offer a novel theory for the spatiotemporal control of molecular flow.

## Results

### Martensitic transition involving reorientation of channels

**Figure 1:**
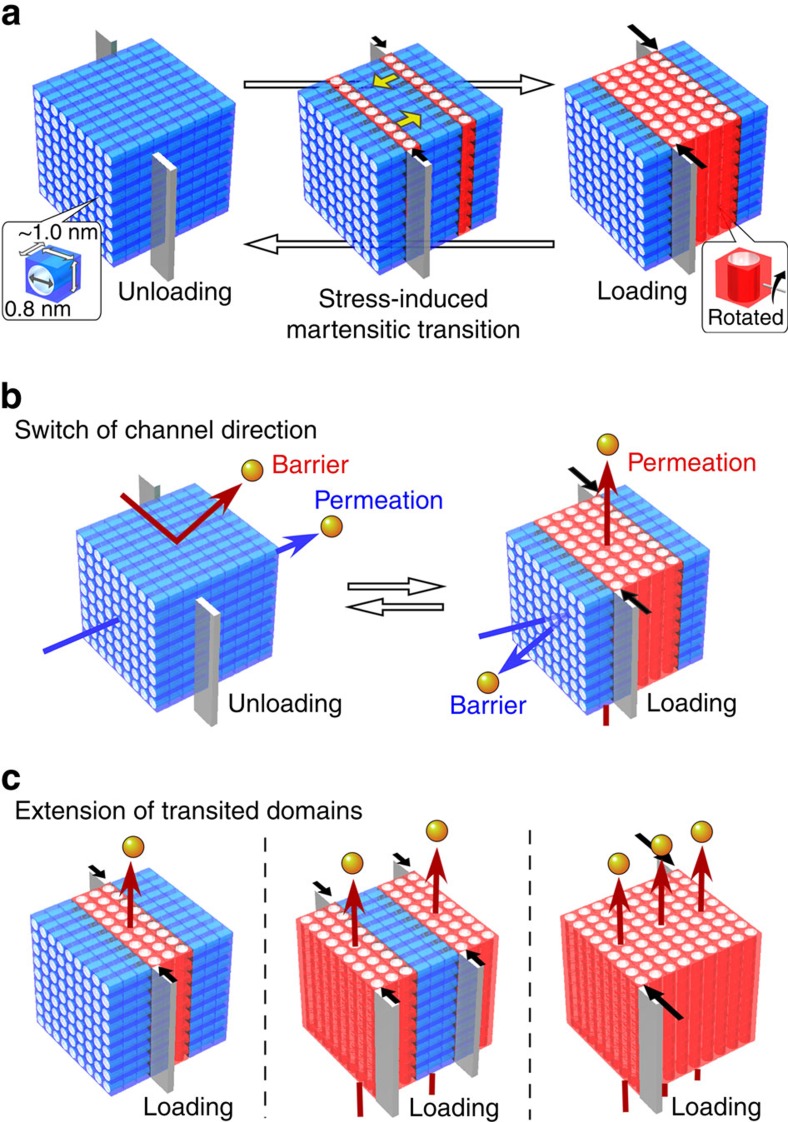
Pore rearrangement in stress-induced transition. (**a**) Rotation of pore directions by generating twinned daughter crystal phase (*α*': red) within mother crystal phase (*α*: blue) during mechanical loading. Expected gas flow switch in direction (**b**) and in positions (**c**) where the area determines the rate of flow mass through subnanometre uniform channels.

**Figure 2:**
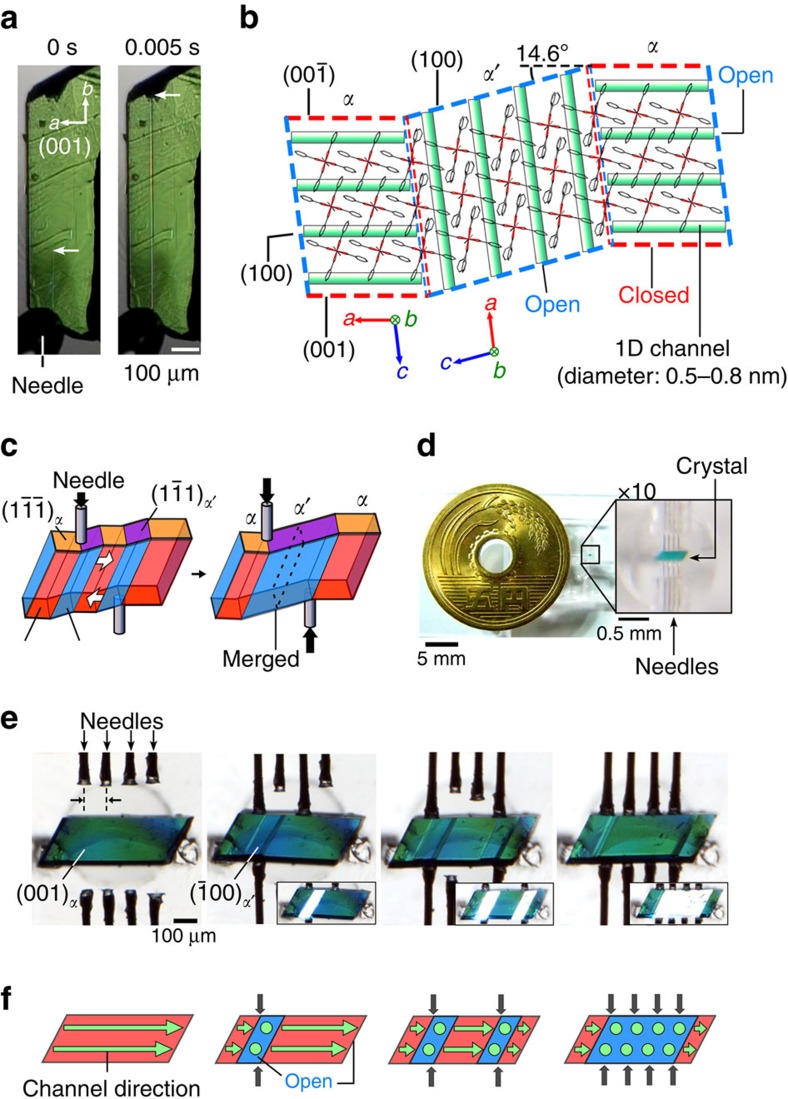
Active positional controllability of martensitic transition. (**a**) Growth of 5-μm-wide band from the pushed edge of the (1-1-1) crystal surface at room temperature. (**b**) Connection of mother (*α* phase) and daughter (*α*' phase) crystals at 298 K accompanied by the rotation of channel direction (green bands) under the twinned state with a bending angle of 14.6° along the projected direction of [010]_*α*_ and [010]_*α*'_ based on crystallography. (**c**) Schematic explanation for the regulation of the *α*' crystal domains sandwiched by the pushing positions as a shear on {1-1-1}_*α*_. (**d**) Picture of an experimental system. (**e**) Active generation/degeneration of daughter crystal domains by shearing the microcrystal of **1** (0.48 × 0.17 × 0.10 mm) with movable needles (**f**) and figures indicating the directions of the penetrating channels. (Inset pictures in **e**: highlighted *α*' domains by reflecting light due to the parallel crystal surfaces in each crystal phase.; see Supplementary Movie 1 for **e**).

We accidentally found stress-induced martensitic transition behaviour on a microporous single-crystal host, [Cu(II)_2_(bza)_4_(pyz)]_*n*_ (bza: benzoate; pyz: pyrazine) (**1**), which can be a microfluidic crystal for gaseous fluid (Fig. 1). By pushing the crystal surface (00-1) of **1** with a glass needle at room temperature, while one edge of the crystal is fixed to a base, a stress-induced daughter phase began to grow out from {1-1-1} with an unchanging bandwidth of about 5 μm sandwiched by two parallel planar interfaces. The edge of the band ran 133 μm ms^−1^ along [010] direction (Fig. 2a). After going across the crystal, the thin band broadened in 0.5 μm ms^−1^ to separate the interfaces (Supplementary Fig. 1). The daughter domain spontaneously contracted and disappeared by removal of the stress through the reverse transition with organosuperelasticity^15^,^16^, which simplifies the reverse operation. This is the first example of a superelastic crystal consisting of metal complexes. Crystal phase indexing under the coexisting state of the mother (*α*) and daughter (*α*′) phases revealed a rotation twin in which the crystal lattice was maintained but was rotated accompanied by the rotation of channel direction in rearranging 0.8-nm-width pore units during the structural phase transition on the boundary (Fig. 2b, Supplementary Figs 2–4 and Supplementary Table 1). The channels in the *α* and *α*′ phases run in a skewed position in the twinned crystal, which is slightly bent at the phase boundary by 14.6° along the projected direction of [010]_*α*_ and [010]_*α*′_, as seen in Fig. 2b. Thus, the generation of the *α*′ phase can change the direction of gas permeation by mechanical twinning and the width or number of channels in the generated *α*′ domains are precisely regulated by the shear range of the mother *α* crystal, as shown in Fig. 2c–f and Supplementary Fig. 5.

### Gas permeability under twinning state

**Figure 3:**
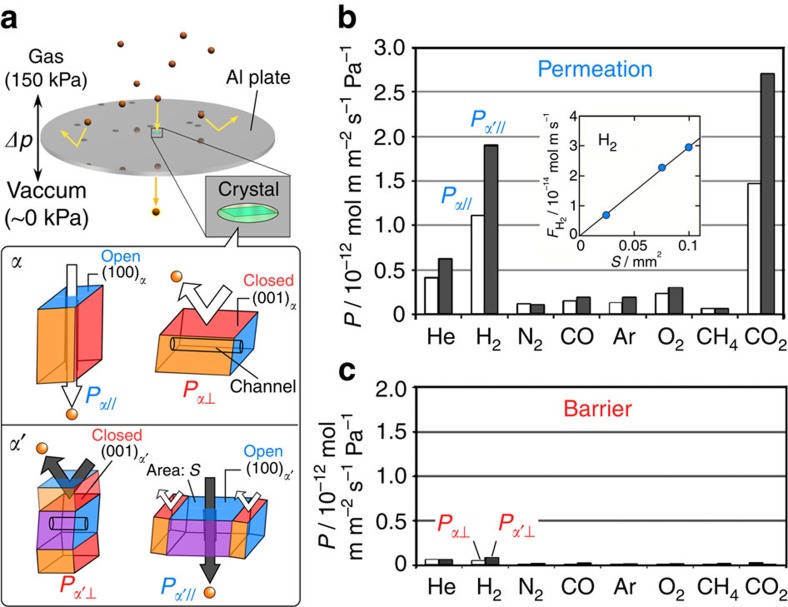
Gas permeation on a single-crystal membrane. (**a**) Schematic explanation for single-crystal membrane and orientations of the embedded crystals in a hole of an Al plate. Gas permeability (*P*) in *α* (white bars) and in *α*' phase (black bars) through open surface (**b**) and closed surface (**c**) of the crystal at 293 K and *Δp* of 150 kPa. (Inset figure in **b**: correlation between open surface area in *α*' phase (*S*) and flow rate of H_2_ gas normalized by crystal thicknesses (*F*_H2_).) The permeability of H_2_ and CO_2_ in the *α*' phase become higher than those in the *α* phase due to the slight change in channel structure caused by molecular distortion (see Supplementary Fig. 7).

We confirmed the interchange of gas flow direction and the regulation of flow rate by crystal twinning of **1** by a gas permeation technique on a single-crystal membrane (Fig. 3a). In the *α* phase crystal where nothing was operated, gases permeate the crystal surfaces of {100}_*α*_ (indicated as the open surface), whereas they were effectively blocked on {001}_*α*_ (indicated as the closed surface), agreeing with the previous report^10^ (white bars in Fig. 3b,c). In the mechanically generated *α*′ phase within the *α* phase crystal, the permeation/barrier directions were interchanged by the reorientation of channel directions estimated in crystallography discussed in Fig. 2b (black bars in Fig. 3b,c). In addition, the extension of the open surface area by widening the *α*′ domain linearly increased the flow rate of H_2_ gas (inset of Fig. 3b), which showed that the number of the channels precisely determines the flow rate due to the identical microflux through the uniform subnanometre channels. Therefore, a finer flux control in molecular scale can be realized by minimizing the *α*′ domain to a nanometre scale or by using additive slits or masks on a crystal if considering the practical limit of the minimum size of a well-controlled domain (5 μm in width in the current crystal membrane).

### Active gas flow control

**Figure 4:**
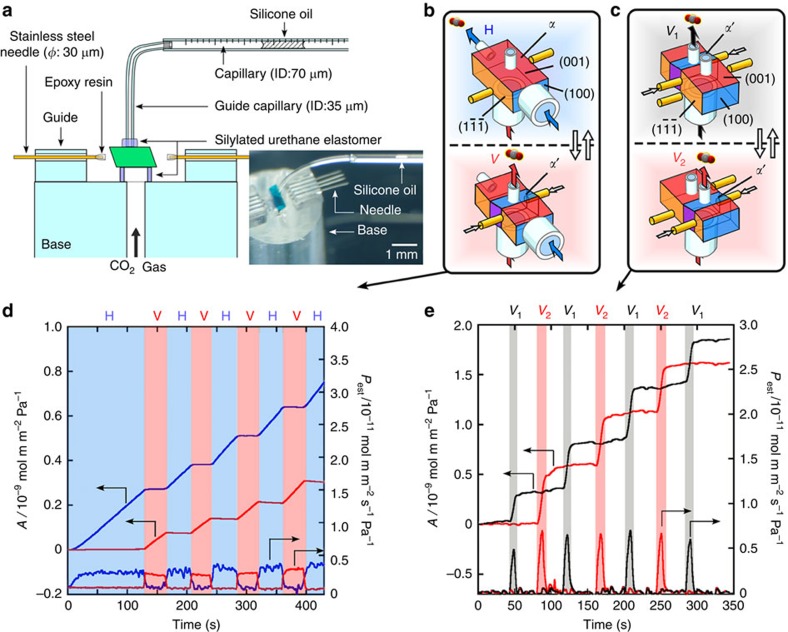
Dynamic gas flow switch. (**a**) Experimental system of spatiotemporal gas permeation measurement with a shearing procedure at room temperature in continuous introduction of CO_2_ gas from the base at *Δp*_CO2_ (difference of partial pressure) of 100 kPa, as shown in Fig. 2e. Schematic explanation for gas flow switch in directions (**b**) and positions of the crystal (**c**). Permeated amount of CO_2_ gas (*A*) and estimated permeability (*P*_est_) through the crystal in H (blue line) and V (red line) directions (**d**) and through two positions (*V*_1_ and *V*_2_) in the V direction (black and red lines) (**e**). (*P*_est_ were estimated as time-derivative values of *A*; Supplementary Movies 2 and 3).

To demonstrate the dynamic switch of the permeated gas flow at the designed time and positions on the crystal **1**, we measured spatiotemporal gas permeation, which was traced by the movement of silicone oil inside capillaries guided through the capillaries attached to the crystal surfaces (Fig. 4a, Supplementary Figs 8 and 9 and Supplementary Movies 2 and 3). CO_2_ was selected in this experiment because of the advantages offered by its permeability among the gases, as summarized in Fig. 3. The directions of gas flow through the crystal specimen were defined as horizontal (H) and vertical (V) derived from crystal orientation (see Fig. 4b). In the *α* phase crystal, gas flowed out from the open surface in the H direction, whereas it was blocked on the closed surface in the V direction (blue region in Fig. 4d). By applying shear stress on the crystal, the permeation/barrier directions were dynamically interchanged in generating *α*′ crystal domain through the rotation of the channel direction (red region in Fig. 4d). The gas permeation starts immediately after *α*′ phase appears (Supplementary Figs 11–16 and Supplementary Tables 6–10). The interchange was spontaneously switched back by removal of the shear stress and could be reproduced repeatedly (Fig. 4d). Applying shear stress at multiple positions dynamically generated the gas flow positions in the crystal (Fig. 4c), which was demonstrated by alternate gas permeations at positions *V*_1_ and *V*_2_, depending on the switch of the sheared positions (black and red regions in Fig. 4e). Therefore, a single crystal of **1** provides spatiotemporal controllability of molecular flow by mechanical twinning.

## Discussion

With respect to the controllability of molecular flow, a single-crystal host of **1** can be regarded as an assemblage of pore units, which can be called ‘porons (pore+on (s))' as they are quasi-particles, with transformability assisted by flexible host skeletons. Dynamic rearrangement of ‘porons' can alter the pore connections, which produces the spatiotemporal controllability of flow directivity within a single solid. In this study, organosuperelasticity is an optimal property for the rearrangement of ‘porons', although other techniques to have an effect on assembly may be available. This theory raises a novel strategy for constructing microfluidic devices.

We have demonstrated the active switchability of gas flow in directions and positions through a microporous molecular crystal of **1** at a designed time caused by stress-induced martensitic transition. The generated daughter domain in mechanical twining involved rotation of the channel directions. The domain spontaneously contracted and disappeared by removal of the stress as a superelasticity, which is the first example in metal complexes. In fact, a single crystal of **1** provides spatiotemporal controllability of molecular flow by mechanical twinning. This microporous single crystal would be used as a device with precise flux and/or directional controllability of a molecular flow manipulated by mechanical stress. Furthermore, by using a stress-induced mechanism in a microdevice, flammable fluid such as high-pressure hydrogen gas would be safely controlled due to the lack of need for electric actuation. Consequently, the introduction of stress-induced transition phenomena into channel solids enables dynamic control of molecular flow in the solids.

## Methods

### X-ray single crystal diffraction analysis

Single-crystal X-ray structural analysis of **1** in *α*/*α*′ coexisting state was performed at 298 K on a Bruker Smart APEX CCD (charge-coupled device) area diffractometer (Bruker AXS K.K.) with a nitrogen-flow temperature controller using graphite-monochromated Mo *Kα* radiation (*λ*=0.71073 Å). Empirical absorption corrections were applied using the SADABS programme. The structure was solved by direct methods (SHELXS-97) and refined by full-matrix least-squares calculations on *F*^2^ (SHELXL-97) using the SHELX TL programme package. Non-hydrogen atoms were refined anisotropically; hydrogen atoms were fixed at calculated positions by riding model approximation.

### Gas permeation measurement

Single crystals of **1** in the *α*′ phase were cut to suitable sizes with an area of the crystal surface such as 1.00 × 10^5^ μm^2^ for (100) and thickness of 80 μm along [100], and 4.78 × 10^4^ μm^2^ for (001) and thickness of 185 μm along [001], which were embedded in a hole of each aluminum plate. These crystal membranes were used in gas permeation measurements along [100] and [001] directions in the *α*′ phase (Supplementary Fig. 6 and Supplementary Tables 2–4). The results are displayed as black bars in Fig. 3b,c, respectively, by using a GTR-20XAYU Analyzer (GTR Tech Corporation) at a differential pressure of 150 kPa and 293 K. Gas permeation was monitored by gas chromatography with a thermal conductivity detector on a GC-2014 Gas Chromatograph (Shimadzu Corporation).

### Active gas flow control

Single crystals of **1** (0.33 × 0.19 × 0.15 mm^3^ for the test of Fig. 4d and 0.36 × 0.10 × 0.10 mm^3^ for the test of Fig. 4e) were placed on a cylindrical glass base (diameter: 2 mm; inner diameter: 0.4 mm), which was equipped with a pair of four stainless-steel needles (diameter: 30 μm; spacing of the pushing positions: 100 μm) for applying force to the crystal membrane (Supplementary Table 5). Glass capillaries (inner diameter: 35 μm) were attached to the crystal by using silylated urethane elastomers and were also connected to gauge capillaries (inner diameter: 65 or 28 μm) including silicone oil (Shin-Etsu Chemical Co., Ltd) as a probe medium for CO_2_ gas permeation. Permeability was calculated with a differential partial pressure of 100 kPa in the CO_2_/membrane/air system. The movement of silicone oil and martensitic transition were observed separately under two microscopes, while making videos at 60 fps.

## Additional information

**How to cite this article:** Takasaki, Y. & Takamizawa, S. Active porous transition towards spatiotemporal control of molecular flow in a crystal membrane. *Nat. Commun.* 6:8934 doi: 10.1038/ncomms9934 (2015).

**Accession code:** The X-ray crystallographic data reported in this paper have been deposited at the Cambridge Crystallographic Data Centre (CCDC), under deposition number CCDC 1421863 and 1421864. These data can be obtained free of charge from The Cambridge Crystallographic Data Centre via.

## Supplementary Material

Supplementary Information Supplementary Figures 1-16, Supplementary Tables 1-10, Supplementary Methods and Supplementary References

Supplementary Movie 1 Movie for generation/degeneration of daughter crystal domains

Supplementary Movie 2 Movie for Gas flow switch in terms of directions of a microcrystal

Supplementary Movie 3 Movie for Gas flow switch in terms of positions of a microcrystal
