# Supplementary material for: Active porous transition towards spatiotemporal control of molecular flow in a crystal membrane
Source: Nat Commun. 2015 Nov 16;6:8934. doi: 10.1038/ncomms9934 (PMC4660351; doi:10.1038/ncomms9934)
Supplement: Supplementary Information — Supplementary Figures 1-16, Supplementary Tables 1-10, Supplementary Methods and Supplementary References [file ncomms9934-s1.pdf]

Supplementary Figures:

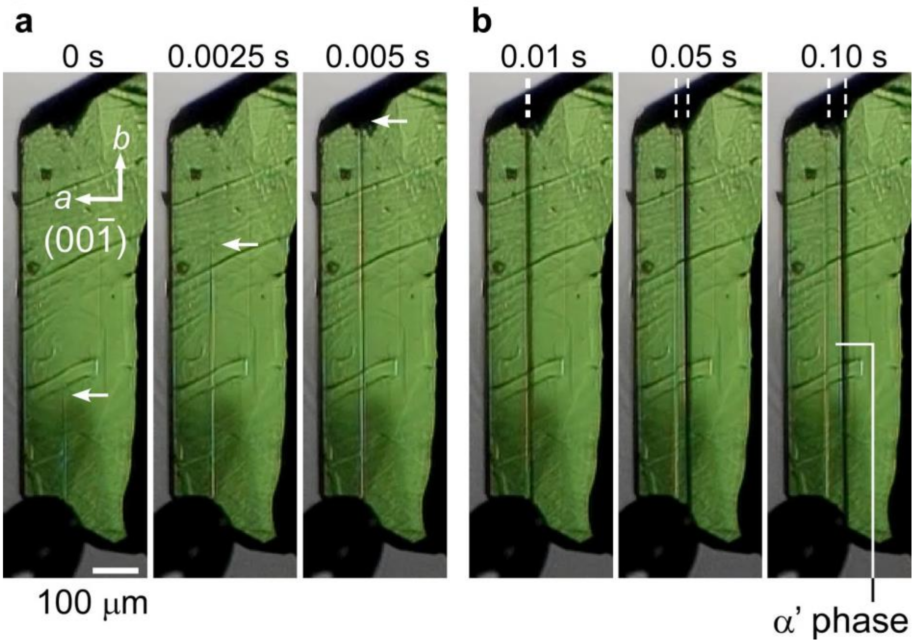

Supplementary Figure 1. Generating processes of twinned daughter (α') phase in mother (α) phase at 298 K: Growth of 5-μm-wide band with a velocity of 133 μm msec^{-1} from pushed edge of (1-1-1) crystal surface (**a**); Transfer of α/α' interfaces with a velocity of 0.5 μm msec^{-1} after forming the band (**b**).

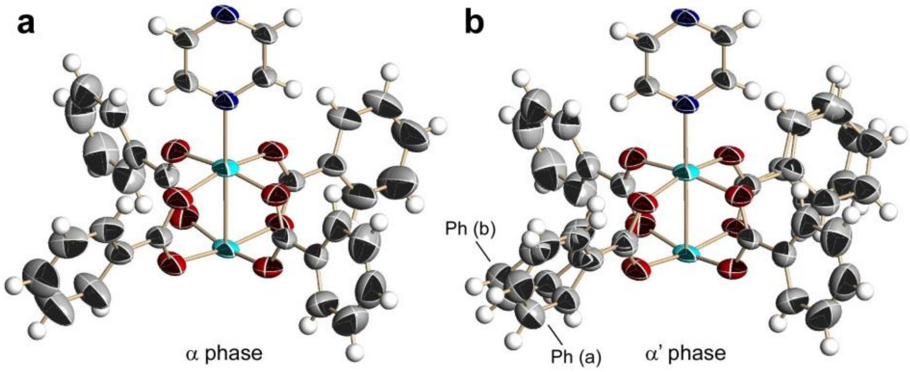

Supplementary Figure 2. Molecular structures of **1** as ORTEP view at 50% probability level for each ellipsoid at 298 K. α phase (**a**) and α' phase with disordered phenyl rings indicated as Ph (a) and Ph (b) (**b**). The occupancy values were refined to 0.7 for Ph (a) and 0.3 for Ph (b).

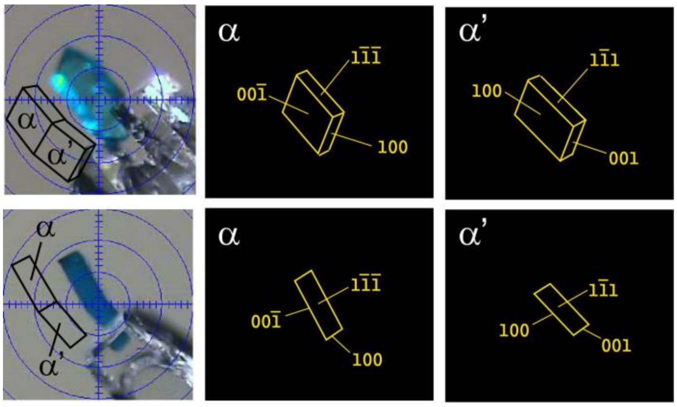

Supplementary Figure 3. Crystal face indexing of **1** in coexisting state of α and α' phase at 298 K. (α/α' interfaces: $(100)_{\alpha}//(00\text{-}1)_{\alpha'}$ or $(001)_{\alpha'}//(-100)_{\alpha}$.)

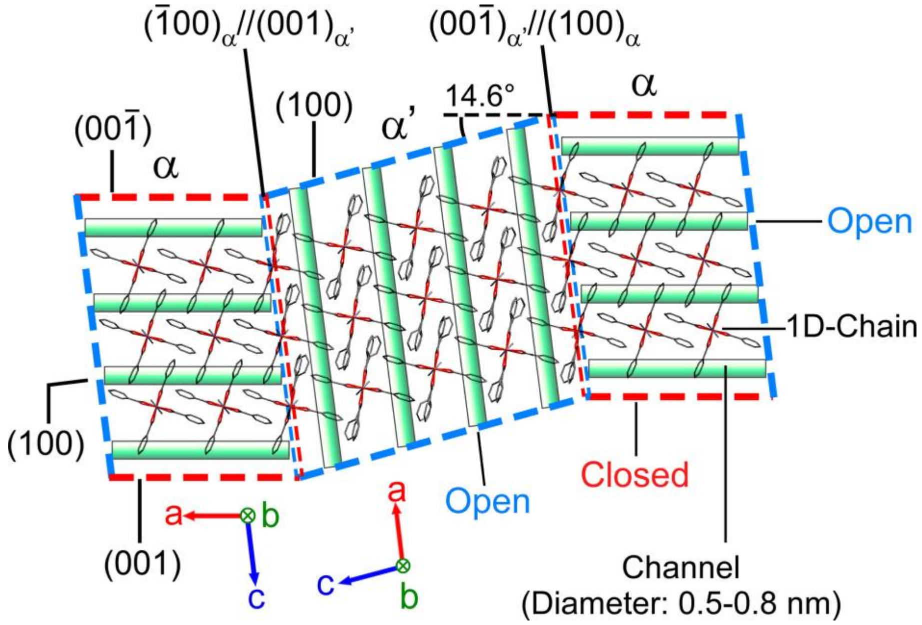

Supplementary Figure 4. Connection of mother (α phase) and daughter (α' phase) crystals under the twinned state accompanied by rotation of channel direction (green bands) with crystal bending by 14.6° along projected direction of $[010]_{\alpha}$ and $[010]_{\alpha'}$, based on crystallography.

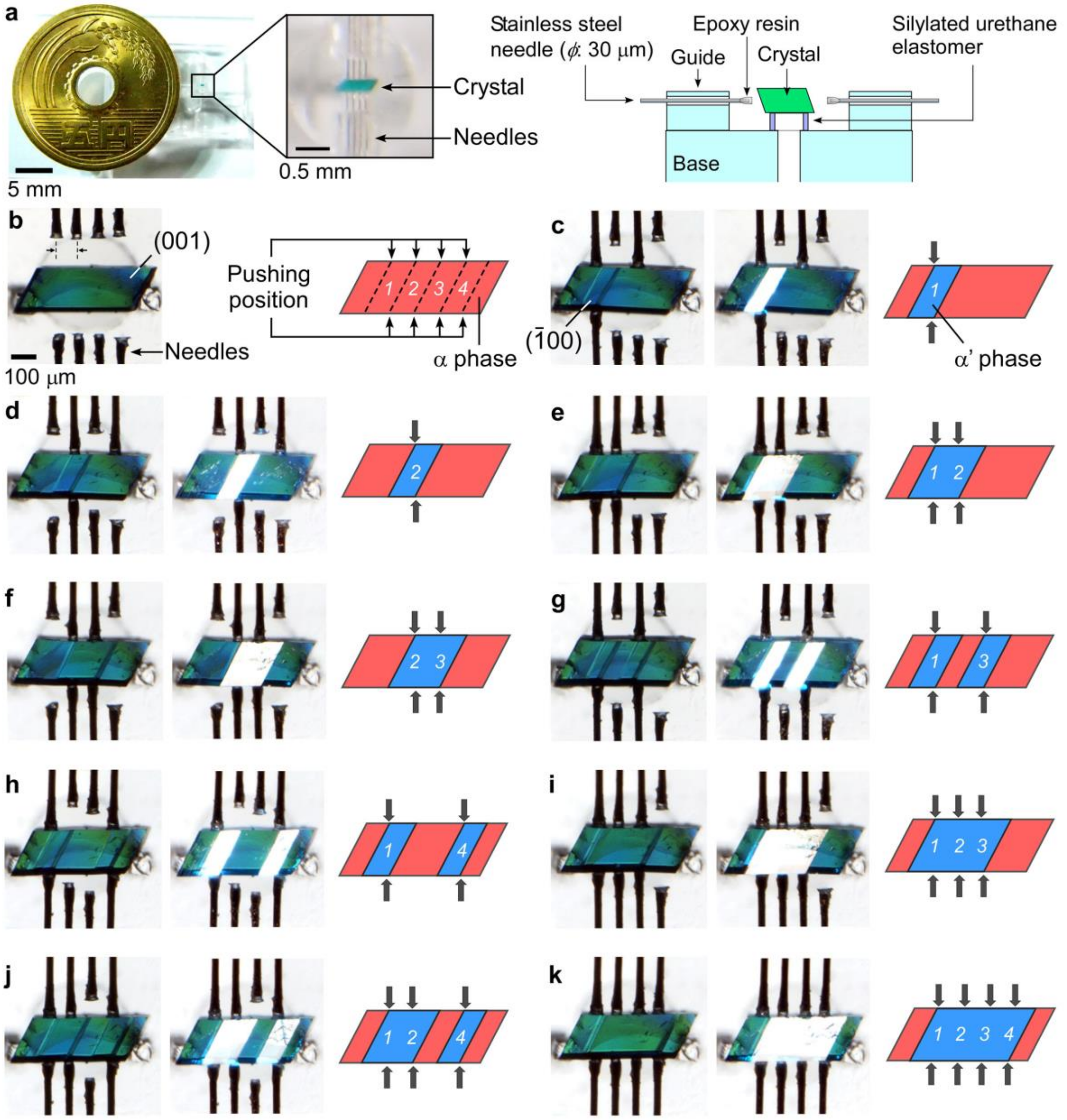

Supplementary Figure 5. Control for positions or widths of generated α' phases in a single crystal. Active generation/degeneration of daughter crystal domains in the microcrystal of **1** (0.48 × 0.17 × 0.10 mm) by shearing on {1-1-1} with movable needles in technically minimum interval of 100 μm. Details of experimental system (**a**); Default single crystal in α phase (left) and schematic figure indicating crystal phase (right) (**b**); Single crystal with generated α' phases in multiple positions and widths (**c**-**k**). (Each right-hand picture: highlighted α' phases by reflecting light due to the parallel crystal surfaces in each crystal phase.) (Supplementary Movie 1)

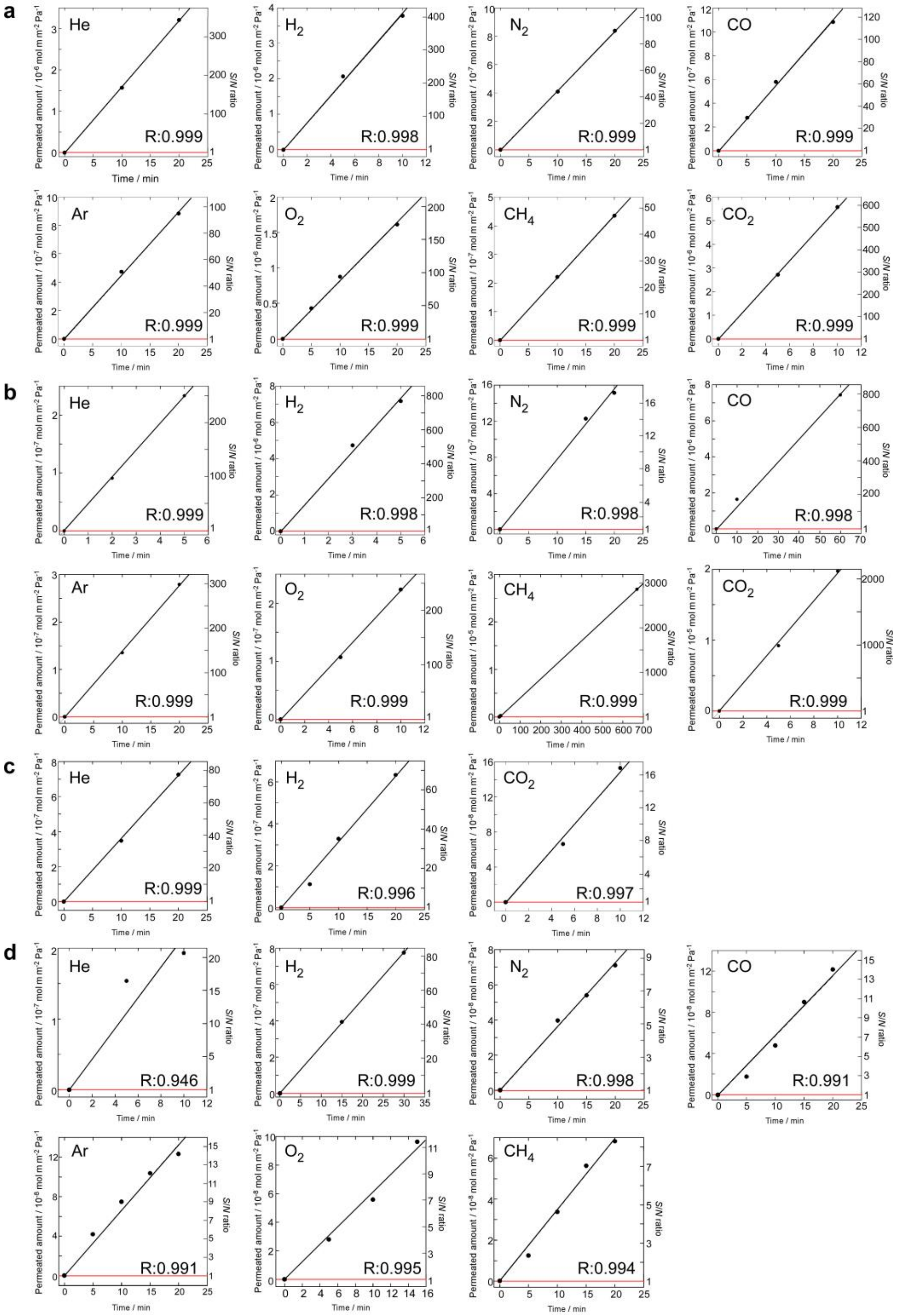

Supplementary Figure 6. Comparison of permeated amount of each gas species through the crystal membrane (left vertical axis) and *S*/*N* ratio of permeation amount versus noise level (right vertical axis). Permeation along the channel in α phase (**a**) and in α' phase (**b**); permeation orthogonal to the channel in α phase (**c**) and in α' phase (**d**). (Red horizontal line: noise level from TCD detector on GC converted as a permeation amount.)

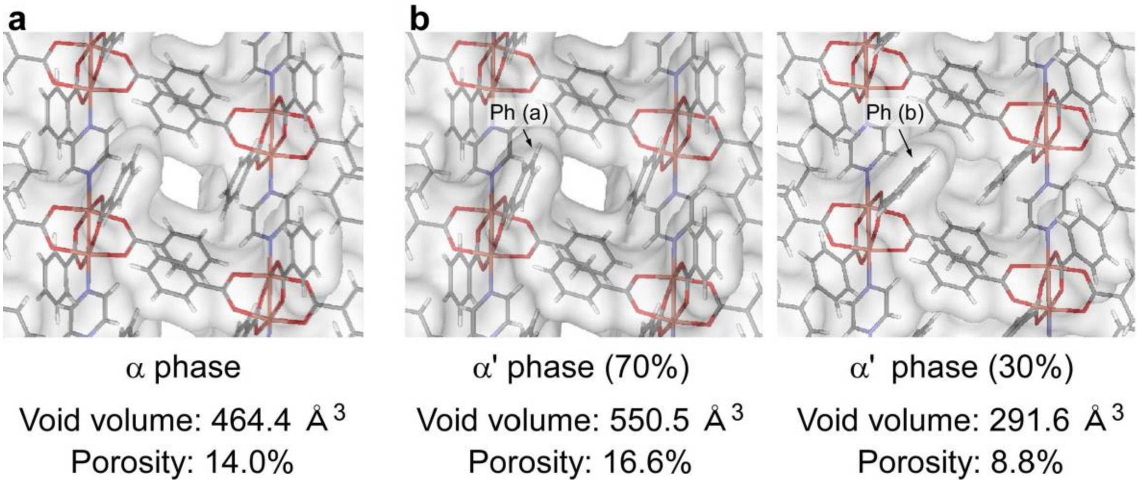

Supplementary Figure 7. Packing structures viewed along the channel direction with superimposed solvent-accessible surfaces (probe radius: 1.4 Å). Structures in α phase (**a**) and in α' phase (**b**) drawn with Ph (a) (left) and with Ph (b) (right). In the α' phase, averaged void volume and porosity were calculated as 472.8 $Å^3$ and 14.3%, respectively, based on their occupancy values. The slight change of molecular orientation can affect gas permeability as shown by the relatively higher permeability of H_2 and CO_2 in the α' phase than those in the α phase.

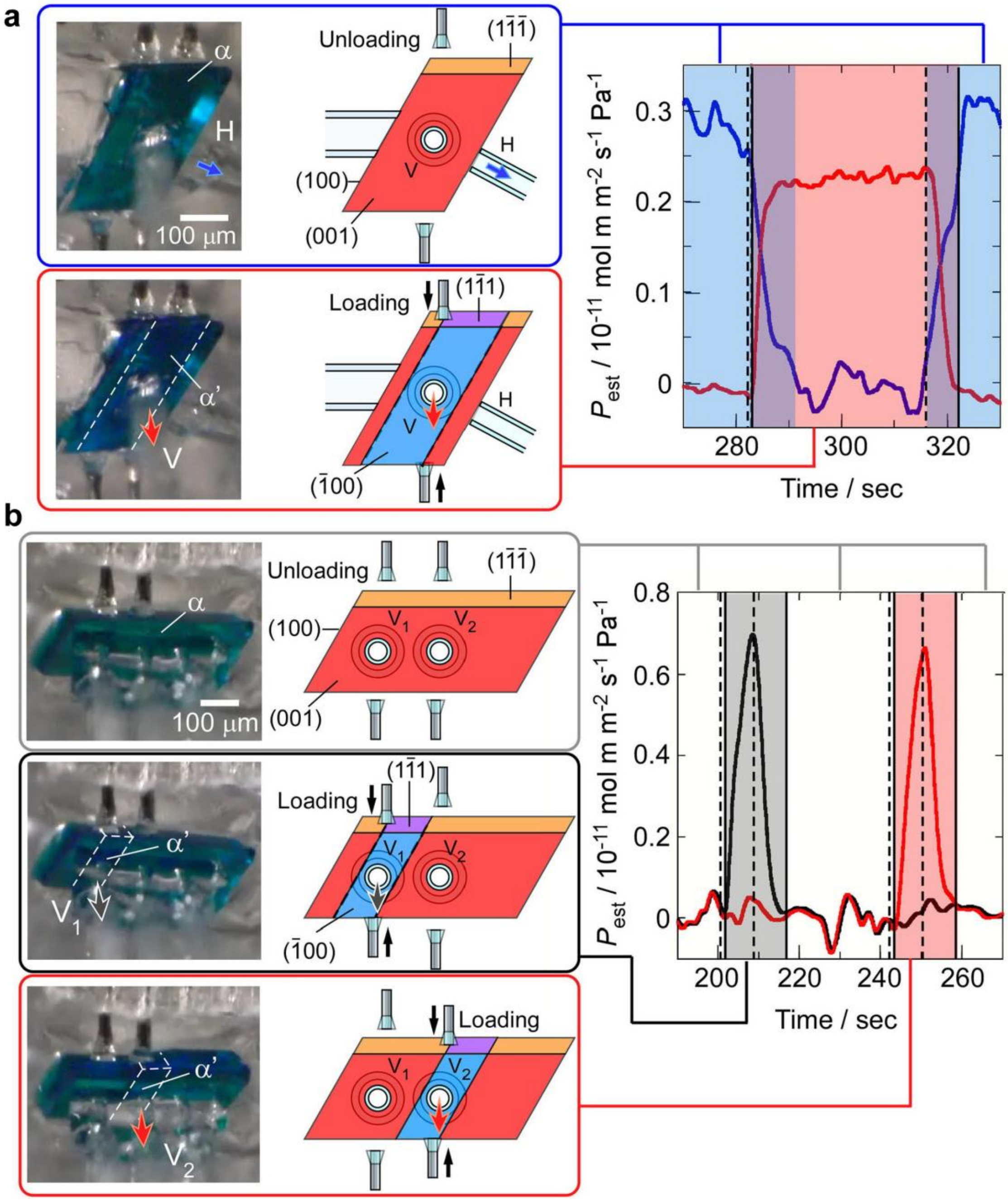

Supplementary Figure 8. Crystal conditions (left) and estimated permeability of CO_2 gas (right) in spatiotemporal gas permeation tests. Gas flow switching in directions (**a**) and in positions (**b**) corresponding to Supplementary Fig. 9. (Dotted lines in right-hand graphs indicate time of applying/removing shear stress.) (Supplementary Movies 2 and 3)

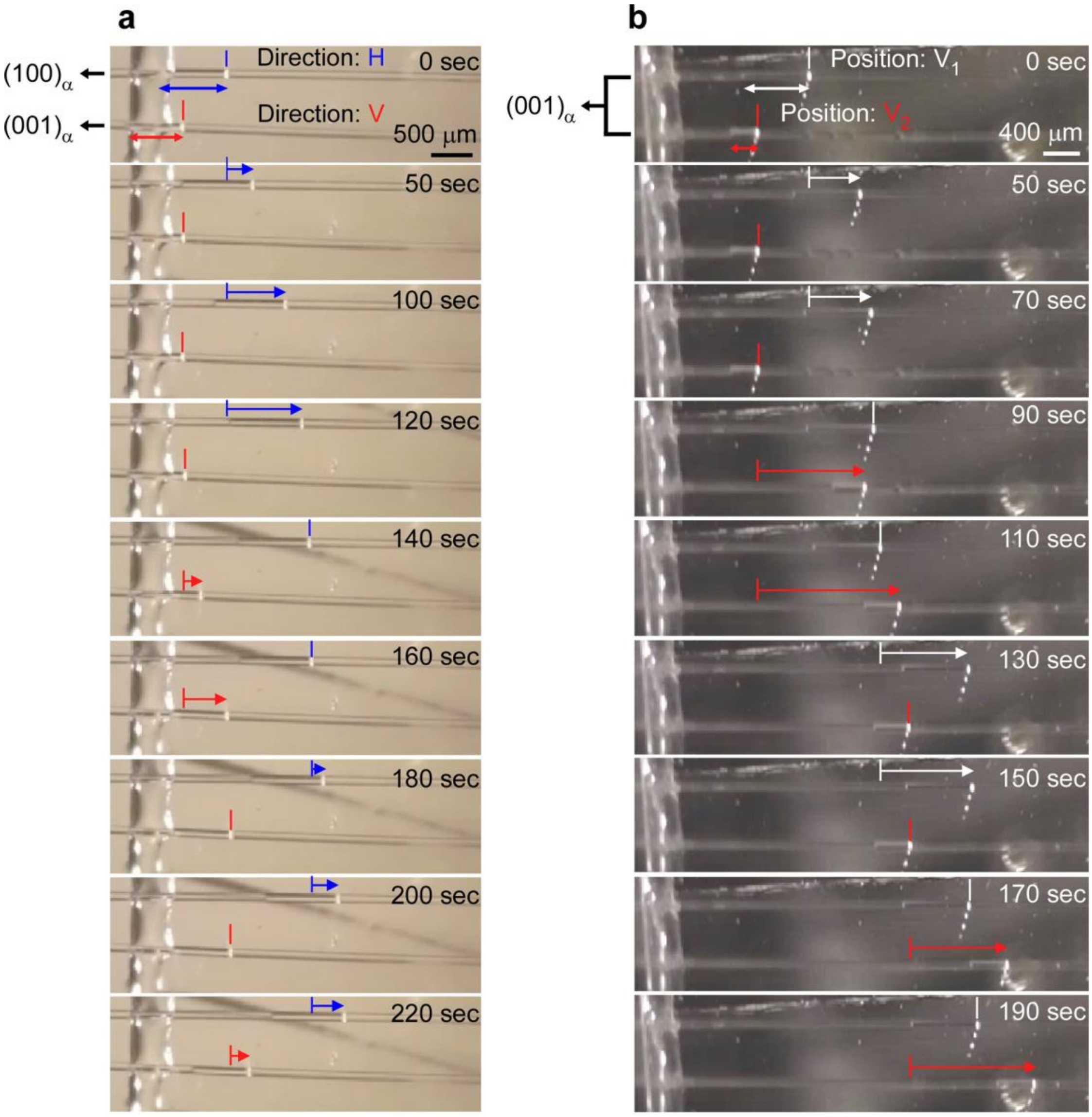

Supplementary Figure 9. Movement of silicone oil inside capillaries pushed out by permeated CO_2 gas from a single crystal of **1**. Traces of gas permeation in V direction (red) and in direction H (blue) (**a**), and gas permeation on position V_1 (white) and V_2 (red) in direction V (**b**) corresponding to Supplementary Figs. 8a and 8b, respectively. (Supplementary Movies 2 and 3)

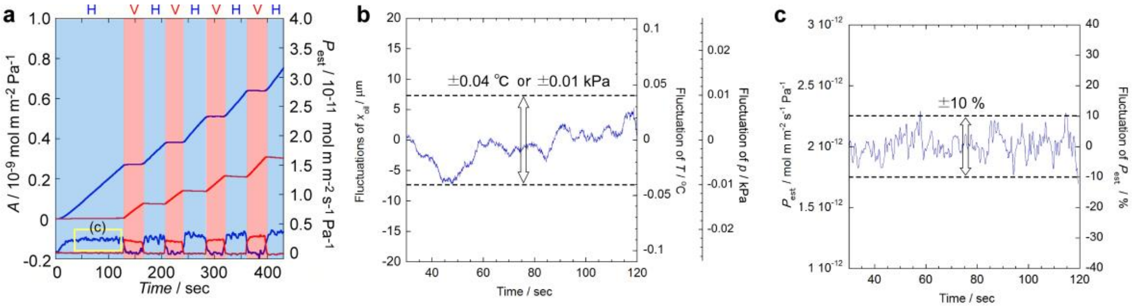

Supplementary Figure 10. Fluctuations of permeability in Fig. 4d affected by temperature or pressure change. Permeation amount (A) and permeability (P_{est}) shown as Fig. 4d of the main text (**a**). Fluctuations of displacement of the oil (x_{oil} / μm: left vertical axis) in the initial steady-state region and the estimated fluctuations of temperature and pressure (T / °C, p / kPa: right vertical axes) calculated by state equation (**b**) (fluctuation of x was obtained by deducting values of a linear line fitted on the actual displacement of the oil. Permeability (P_{est} / mol m m^{-2} s^{-1} Pa^{-1}: left vertical axis) and fluctuations (right vertical axis) (**c**). Such changes per time cause fluctuations of permeability about ±10%. (Steady-state region) However, the considerably small allowance in temperature or pressure indicates the severity of our atmospheric control.

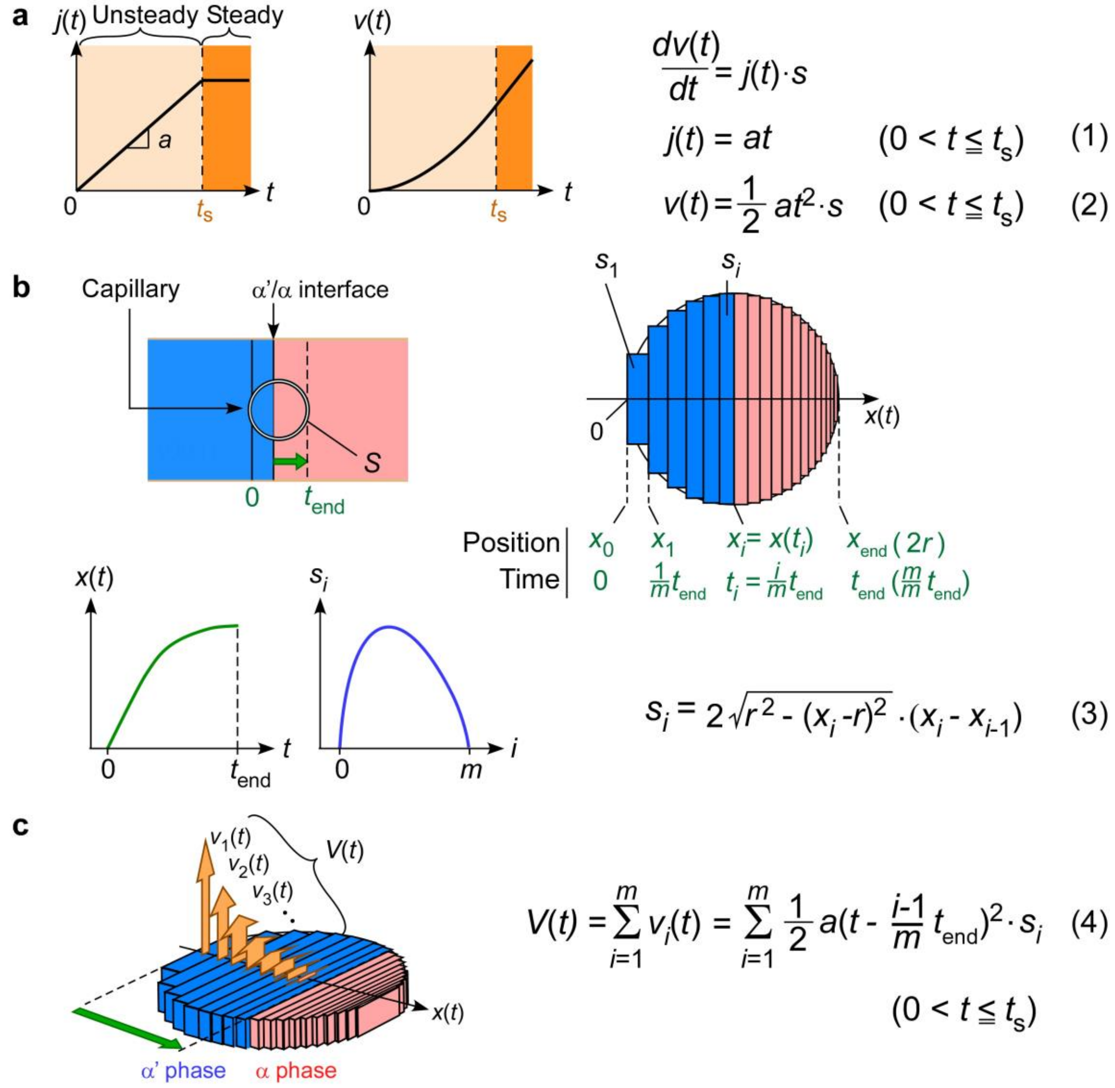

Supplementary Figure 11. Estimation of permeated gas volume during propagation of α' phase. Relationship of flux ($j(t)$) and permeated volume ($v(t)$) through a membrane with permeation area of s (**a**) (t_s means the time to change into steady-state permeation from the unsteady state setting linear slope (a) of flux in the unsteady state). Calculation of divided permeation area (s_i) (**b**) (blue: induced daughter α' phase, red: mother α phase; bottom-left figure: position of interface ($x(t)$); bottom-right figure: divided permeation areas (s_i)). Calculation of permeated gas volume ($V(t)$) until t (**c**).

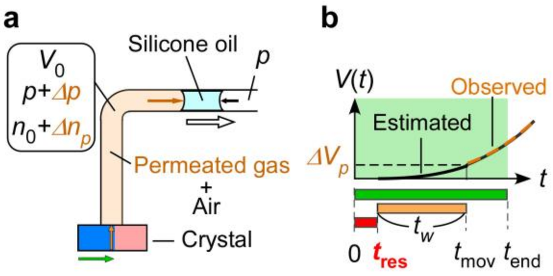

$$t_{res} = t_{mov} - t_w \quad (5)$$

$$\Delta p = \lambda \cdot \frac{L}{d} \cdot \frac{\rho u^2}{2} \qquad \left(\lambda = 64 \frac{\nu}{ud}\right) \quad (6)$$

$$\Delta V_p = V_0 \frac{\Delta n_p}{n_0} \qquad \left(\frac{n_0 + \Delta n_p}{n_0} = \frac{p + \Delta p}{p}\right) \quad (7)$$

Supplementary Figure 12. Calculation of response time (t_{res}) by estimating the waiting time (t_w) required to move silicone oil in a capillary. Initial moment when the oil moves (**a**). Schematic diagram of $V(t)$ versus t (**b**). Parameters in each equation are explained in Supplementary Table 10. Estimated pressures of Δp (0.034-0.21 Pascal) from eq. 6[2] were sufficiently small, enabling calculation of ΔV_p while assuming $p+\Delta p$ = 100 kPa. (Supplementary Tables 6-9)

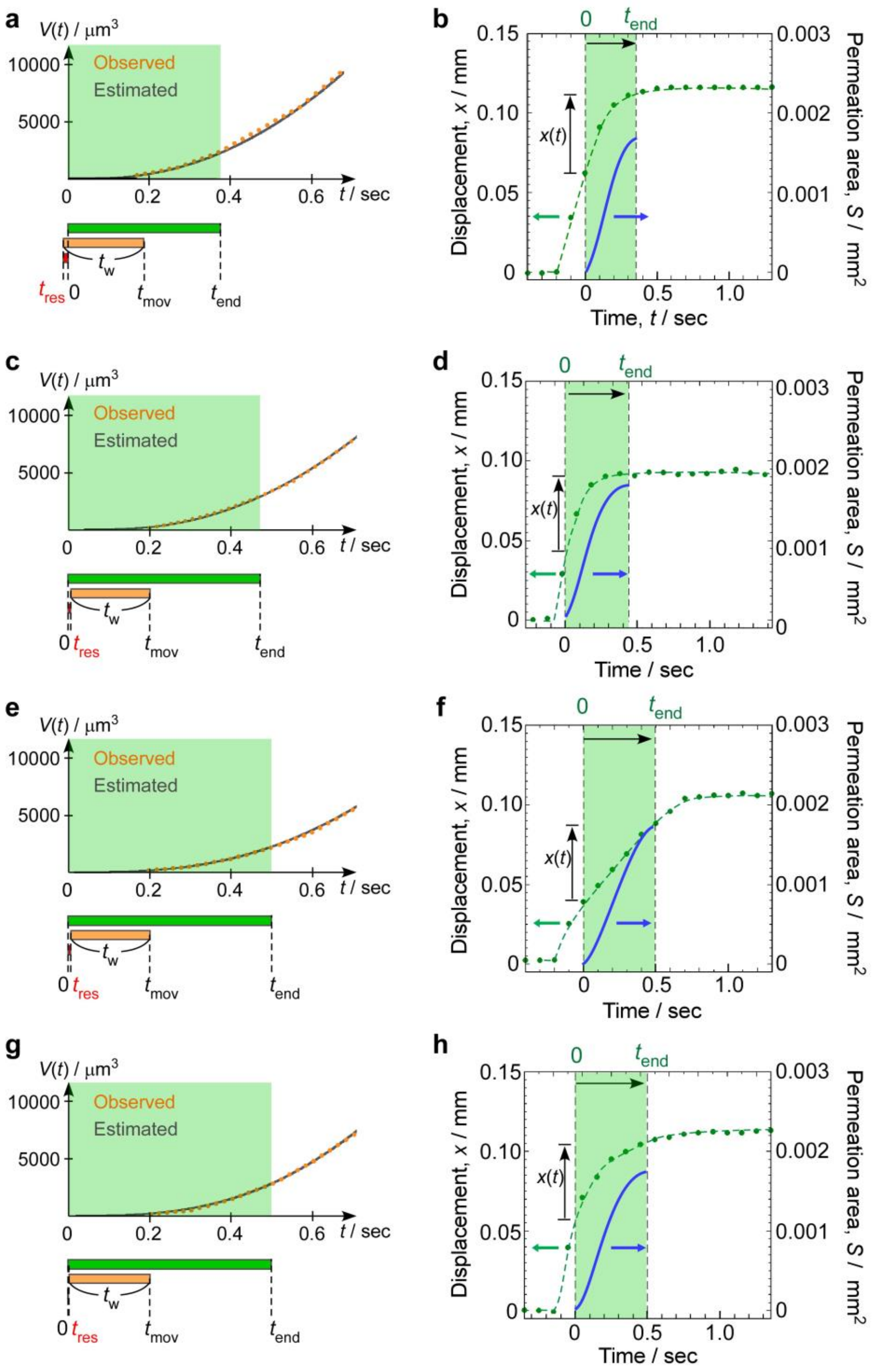

Supplementary Figure 13. Estimation of t_{res} and t_w based on curve fittings of $V(t)$ along the channel in the α’ phase (V direction in Fig. 4d) at the first (128 sec) (**a, b**), second (205 sec) (**c, d**), third (283 sec) (**e, f**), and forth (359 sec) (**g, h**) induction of shear-induced phase transition, as shown in Fig. 4d. (Each left-hand figure: curve fittings of $V(t)$ by eq. 4; each right-hand figure: displacements of moving α’/α interface (x: green plots) observed under a microscope and permeation areas (S: blue lines).)

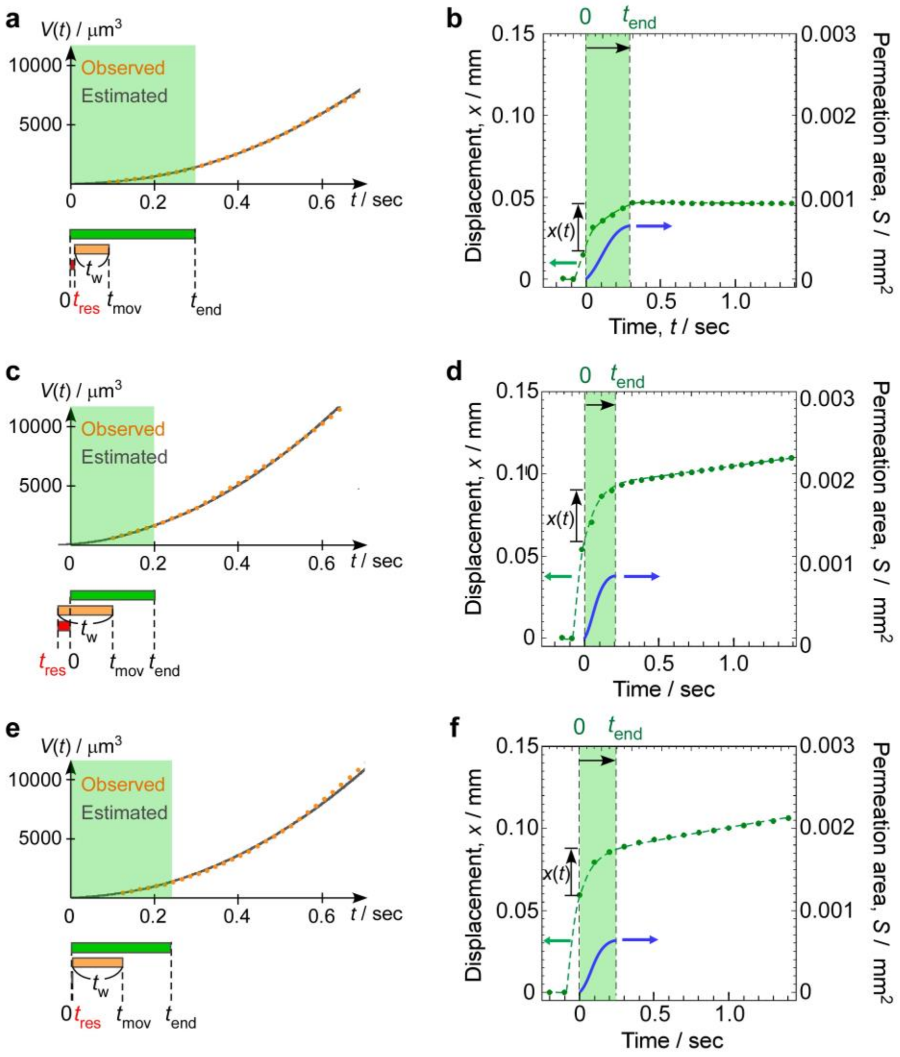

Supplementary Figure 14. Estimation of t_{res} and t_w based on curve fittings of $V(t)$ from two positions (positions V_1 and V_2 in Fig. 4e) on (100) in α' phase at the first (44 sec) (**a, b**), second (81 sec) (**c, d**), and third (117 sec) (**e, f**) induction of shear-induced phase transition, as shown in Fig. 4e. (Each left-hand figure: curve fittings of $V(t)$ by eq. 4; each right-hand figure: displacements of moving α'/α interface (x: green plots) observed under a microscope and permeation areas (S: blue lines).)

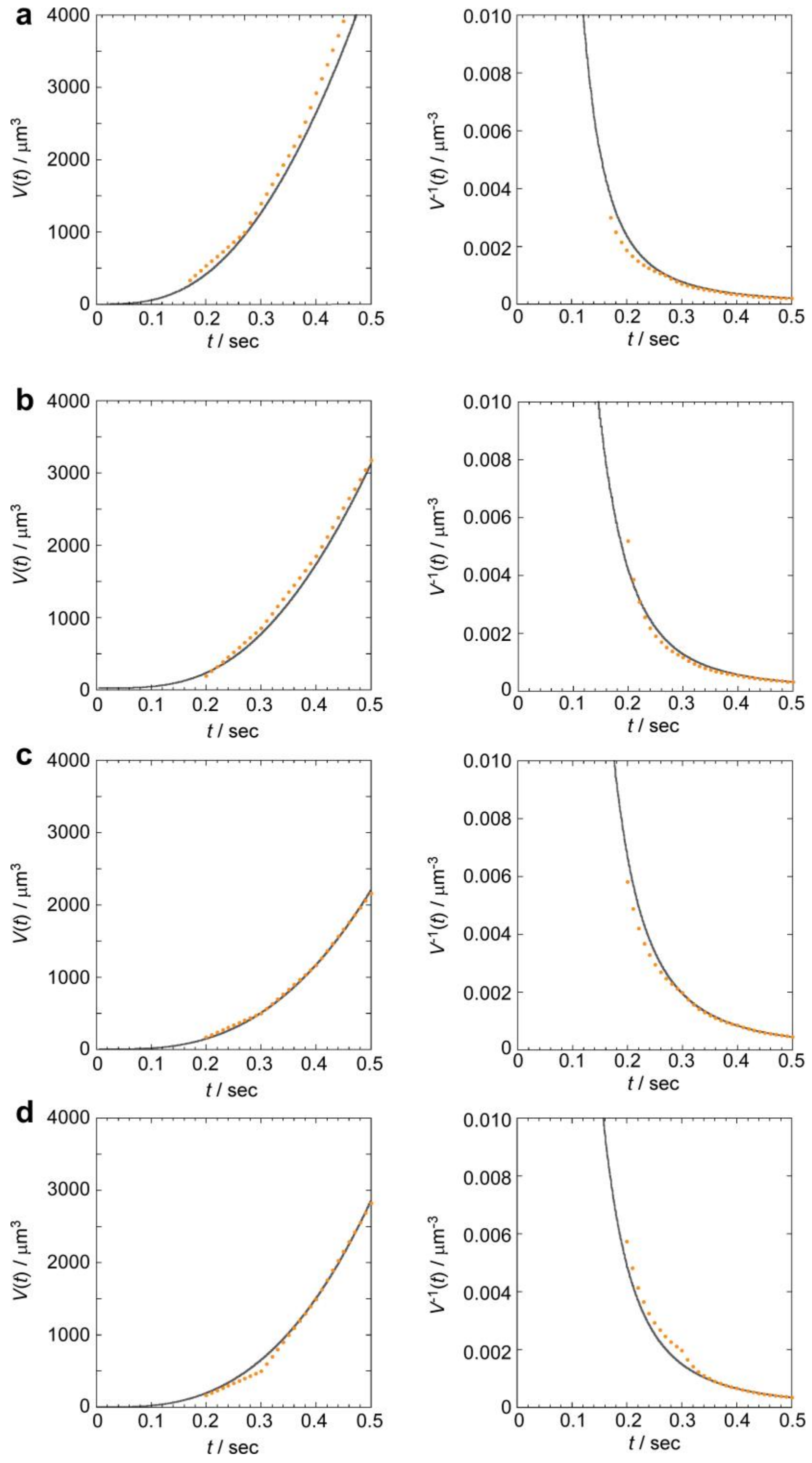

Supplementary Figure 15. Best fitting curves (solid black lines) for normal plots (left) and reciprocal plots (right) focusing on earlier region of $V(t)$ (yellow plots) versus t. **a, b, c,** and **d** correspond to the first to forth induction of shear-induced phase transition at 128, 205, 283, and 359 sec in Fig. 4d, respectively.

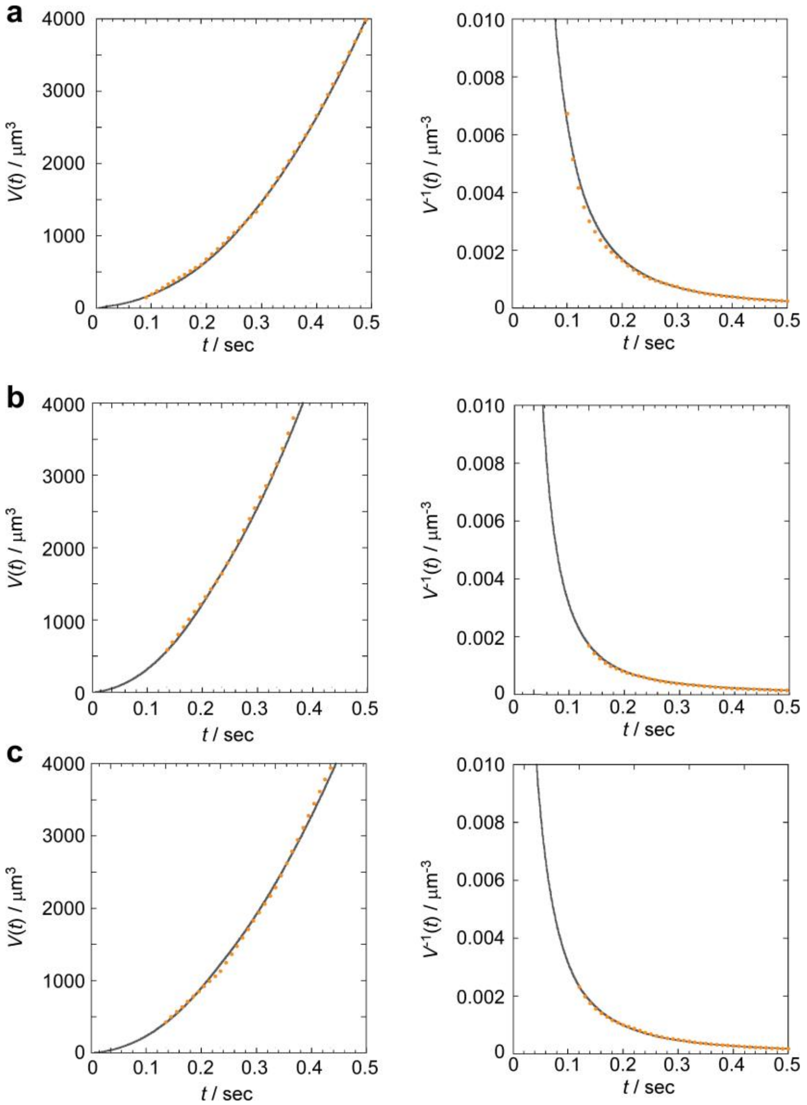

Supplementary Figure 16. Best fitting curves (solid black lines) for normal plots (left) and reciprocal plots (right) focusing on earlier region of $V(t)$ (yellow plots) versus t. **a, b,** and **c** correspond to the first to third induction of shear-induced phase transition at 44, 81, and 117 sec in Fig. 4e, respectively.

Supplementary Tables:

Supplementary Table 1. Crystallographic data of **1** in α and α' phases.

Crystal phase	α	α'
Empirical formula	C_{32} H_{24} Cu_2 N_2 O_8	C_{32} H_{24} Cu_2 N_2 O_8
Crystal size / mm^3	0.17×0.13×0.07	0.18×0.13×0.07
M	691.61	691.61
Crystal system	Monoclinic	Monoclinic
Space group	$C2/c$	$C2/c$
T / K	298	298
a / Å	18.098(2)	18.107(4)
b / Å	9.7040(10)	9.705(2)
c / Å	18.991(2)	18.997(4)
α / deg	90	90
β / deg	97.328(3)	97.294(5)
γ / deg	90	90
V / $Å^3$	3308.1(6)	3311.3(12)
Z	4	4
D_{calcd} / Mg m^{-3}	1.389	1.387
μ(Mo Kα) / mm^{-1}	1.335	1.334
Reflections collected	11966	11966
Independent reflections (R_{int})	2322(0.0543)	2322(0.0543)
Completeness / %	99.5	99.6
Goodness of fit	1.039	1.051
R_1(I > 2σ (all data))	0.0680(0.1170)	0.0681(0.1177)
wR_2(I > 2σ (all data))	0.1951(0.2241)	0.1957(0.2255)
Largest diff. peak (hole) / $eÅ^3$	1.009(-0.509)	1.032(-0.494)

CCDC numbers: 1421863 (α phase) and 1421864 (α' phase).

Supplementary Table 2. List of gas permeability in α phase[1] and α' phase.

Gas	$P_{[100]\alpha}$ [1] / mol m m^{-2} s^{-1} Pa^{-1}	$P_{[100]\alpha'}$ / mol m m^{-2} s^{-1} Pa^{-1}	$P_{[001]\alpha}$ [1] / mol m m^{-2} s^{-1} Pa^{-1}	$P_{[001]\alpha'}$ / mol m m^{-2} s^{-1} Pa^{-1}
He	4.13×10^{-13}	6.20×10^{-13}	5.93×10^{-14}	6.65×10^{-14}
H_2	1.11×10^{-12}	1.90×10^{-12}	4.59×10^{-14}	7.99×10^{-14}
N_2	1.11×10^{-13}	1.06×10^{-13}	nd	1.11×10^{-14}
CO	1.49×10^{-13}	1.90×10^{-13}	nd	1.78×10^{-14}
Ar	1.22×10^{-13}	1.83×10^{-13}	nd	2.04×10^{-14}
O_2	2.29×10^{-13}	2.98×10^{-13}	nd	1.94×10^{-14}
CH_4	5.85×10^{-14}	6.00×10^{-14}	nd	1.07×10^{-14}
CO_2	1.47×10^{-12}	2.70×10^{-12}	2.38×10^{-14}	nd

[a] Permeability was calculated from the permeation amounts in plural setting time at 293 K and differential pressure of 150 kPa.

[b] $P_{[100]\alpha}$, $P_{[100]\alpha'}$: permeability along channel; $P_{[001]\alpha}$, $P_{[001]\alpha'}$: permeability orthogonal to channel.

[c] "nd" means permeability less than the lower detection limit (TCD's noise level) corresponding to 0.5×10^{-14} mol m m^{-2} s^{-1} Pa^{-1}.

Supplementary Table 3. Detailed information of single-crystal membranes.

Crystal phase	Membrane surface	Surface area / μm^2	Membrane thickness / μm
α	(100)	5.35×10^4	160
α'	(100)	1.00×10^5	80
α	(001)	1.73×10^5	100
α'	(001)	4.78×10^4	185

Supplementary Table 4. *S*/*N* ratios and correlation coefficients (R) of gas permeation measurements in Fig. 3.

Gas	$[100]_{\alpha}$[1]	$[100]_{\alpha'}$	$[001]_{\alpha}$[1]	$[001]_{\alpha'}$
He	343 (0.999)	250 (0.999)	77.6 (0.999)	20.6 (0.946)
H_2	403 (0.998)	766 (0.998)	67.7 (0.996)	82.9 (0.999)
N_2	89.8 (0.999)	162 (0.998)	-	7.58 (0.998)
CO	115 (0.999)	793 (0.998)	-	14.0 (0.991)
Ar	94.7 (0.999)	298 (0.999)	-	14.1 (0.991)
O_2	173 (0.999)	239 (0.999)	-	11.3 (0.995)
CH_4	46.5 (0.999)	2874 (0.999)	-	8.3 (0.994)
CO_2	597 (0.999)	2110 (0.999)	16.8 (0.997)	-

[a] *S*: Permeation amount of the last plots; *N*: noise level converted as a permeation amount.

[b] Values in parentheses are correlation coefficients (R) of least square fittings.

[c] Hyphen means none of the observed permeation amount due to the *S*/*N* ratio is less than 1.

Supplementary Table 5. Conditions of spatiotemporal gas permeation tests.

Temperature / °C	Crystal dimension / mm^3	Surface area (crystal surface) / μm^2	Thickness (direction) / µm	Inner diameter of capillary / µm	Shearing surface
24	0.33 × 0.19 × 0.15	1.71×10^3 (001);<br>9.08×10^2 (100)	150 ([001]);<br>190 ([100])	65 µm	{1-1-1}
25	0.36 × 0.10 × 0.10	6.17×10^2 (001)<br>7.85×10^2 (001)	104 ([001])	28 µm	{1-1-1}

Supplementary Table 6. Values used for calculating t_{res} in experiment of active gas flow control.

	l / µm	S / µm^2	x_{end} / µm	d / µm	p_d / kPa	v / mm^2 s^{-1}	ρ / kg m^{-3}	L / mm
(1)	150	1.71 × 10^3	46.6 (V)	65	101.3	100	0.965 × 10^3	0.0029
(2)	104	6.17 × 10^2	28.0 (V_1)	28	101.3			0.0095
(3)		7.85 × 10^2	31.6 (V_2)					

[a] Values of (1), (2), and (3) correspond to the fittings in Supplementary Figs. 15a-d, Figs. 16a,c, and Fig. 16b, respectively.

Supplementary Table 7. Calculated viscous resistance (bold), reduced gas volume (bold), and related parameters (plain) at the initial moment of oil transfer.

	**Δp / kPa**	**ΔV_p / µm^3**	u / mm s^{-1}	V_0 / µm^3
(1)	**3.6 × 10^{-5}**	**63.0**	0.017	1.77 × 10^8
(2)	**3.4 × 10^{-5}**	**59.4**	0.016	
(3)	**3.8 × 10^{-5}**	**66.5**	0.018	
(4)	**4.0 × 10^{-5}**	**70.0**	0.019	
(5)	**1.2 × 10^{-4}**	**206**	0.095	1.74 × 10^8
(6)	**2.1 × 10^{-4}**	**362**	0.169	
(7)	**1.7 × 10^{-4}**	**293**	0.135	

[a] $\Delta p = \lambda L\rho u^2/2d$ ($\lambda = 64v/ud$) (eq. 6); $\Delta Vp = V_0 \Delta n_p/n_0$ (($n_0+\Delta n_p)/n_0 = (p+\Delta p)/p$) (eq. 7)

[b] Values of (1-4) and (5-7) correspond to the fittings in Supplementary Figs. 15a-d and 16a-c, respectively.

Supplementary Table 8. Calculated (bold) and observed (plain) values of parameters in best fitting of Supplementary Figs. 13 and 15 for determining t_{res}.

	**t_{res}** / s	t_{mov} / s	**t_w / s**	t_{end} / s	t_s / s	**a / mol m^{-2} s^{-2}**	**P_{CO2} / mol m m^{-2} s^{-1} Pa^{-1}**	ΔV_p / µm^3
(a)	**-0.010**	0.17	**0.180**	0.37	1.3	**1.41 × 10^{-3}**	**2.71 × 10^{-12}**	259
(b)	**0.005**	0.20	**0.195**	0.47	1.4	**1.27 × 10^{-3}**	**2.63 × 10^{-12}**	211
(c)	**0.006**	0.20	**0.194**	0.50	1.7	**1.15 × 10^{-3}**	**2.89 × 10^{-12}**	145
(d)	**0.001**	0.20	**0.199**	0.50	1.6	**1.69 × 10^{-3}**	**4.00 × 10^{-12}**	196

[a] $t_{res} = t_{mov} - t_w$ (eq. 5); $P_{CO2} = a \cdot t_s \cdot l \cdot p_d^{-1}$ (P_{CO2} in Fig. 3b: 2.70 × 10^{-12} mol m m^{-2} s^{-1} Pa^{-1}.)

[b] Values of (a-d) correspond to the fittings in Supplementary Figs. 13a, c, e, g, and 15a-d.

Supplementary Table 9. Calculated (bold) and observed (plain) values of parameters in best fitting of Supplementary Figs. 14 and 16 for determining t_{res}.

	**t_{res}** / s	t_{mov} / s	**t_w / s**	t_{end} / s	t_s / s	**a / mol m^{-2} s^{-2}**	**P_{CO2} / mol m m^{-2} s^{-1} Pa^{-1}**	ΔV_p / µm^3
(a)	**0.010**	0.10	**0.09**	0.30	3.5	**1.81 × 10^{-3}**	**6.50 × 10^{-12}**	157
(b)	**-0.030**	0.10	**0.130**	0.20	3.0	**1.90 × 10^{-3}**	**5.85 × 10^{-12}**	573
(c)	**0.001**	0.12	**0.119**	0.24	2.9	**2.20 × 10^{-3}**	**6.55 × 10^{-12}**	429

[a] $t_{res} = t_{mov} - t_w$ (eq. 5); $P_{CO2} = a \cdot t_s \cdot l \cdot p_d^{-1}$ (P_{CO2} in Fig. 3b: 2.70 × 10^{-12} mol m m^{-2} s^{-1} Pa^{-1}.)

[b] Values of (a-c) correspond to the fittings in Supplementary Figs. 14a, c, e, and 16a-d.

Supplementary Table 10. Explanations of valuables and parameters.

t_{res}	Estimated response time.
t_s	Time required to change into steady-state permeation from unsteady state.
t_{end}	Time of the interface departing the capillary.
t_w	Waiting time required to move silicone oil in a capillary.
t_{mov}	Initial time observing oil movement.
m	Dividing number of permeation area in a sectional measurement.
$x(t)$	Position of moving interface at t.
x_{end}	Position of moving interface at t_{end}, which is equal to diameter of capillary ($2r$).
$j_i(t)$	Flux of permeated gas from i-th divided region at t.
$v_i(t)$	Gas volume permeated from i-th divided region until t.
V_0	Initial inner volume of capillary between crystal surface and inside-end of capping oil.
ΔV_p	Estimated gas volume until t_{mov} reduced from Δn_p at 100 kPa.
$V(t)$	Total gas volume from crystal membrane until t.
n_0	Default amount of substance (air) in capillary.
Δn_p	Permeated amount of substance (CO_2) during $t_{w.}$
p	Default pressure in capillary (100 kPa).
Δp	Additional pressure to move oil.
p_d	Differential partial pressure between top and bottom surface of crystal membrane.
s_i	Permeation area on i-the divided region.
S	Total permeation area under capillary attached to the crystal surface.
l	Crystal thickness.
P_{CO2}	Estimated permeability of CO_2 gas based on fitting parameters.
λ	Resistance factor in pipe (capillary) including fluid.
L	Virtual distance of moving oil until t_{mov} at velocity of u.
d	Inner diameter of capillary including oil.
ρ	Density of fluid (silicone oil).
u	Velocity of fluid in steady state.
v	Kinetic viscosity of fluid at specific temperature. (Value of silicone oil at 298 K)

Supplementary Methods:

Estimation of gas permeability values.

The gas permeability values in Fig. 3 and Supplementary Table 2, which were taken from one single crystal membrane, have reproducibility because the permeation amount for every gas species increased in proportion to the setting time in the cycling measurements at 0-5, 0-10, 0-15, and 0-20 min, for examples. Therefore, the unique slope of the line on each plot (correlation coefficient: R = 0.946-0.999) gives a reliable value of permeability, which secondarily depends on the signal/noise ratio from a TCD detector on GC. (Supplementary Table 4)

Response time in active gas flow control.

The response time (t_{res}) of gas permeation after the α' phase appears was estimated by the time course of the permeated gas volume ($V(t)$) during propagation of the mechanically induced phase, which indicates that the gas permeation starts immediately after the transition. (Thus, t_{res} is zero, and the permeated amount is small even though the value of t_{res} is small.)

By assuming the slope of increasing flux ($j(t)$) as a constant (a), (Supplementary Equation 1) permeated gas volume ($v(t)$) through the membrane area (s) until t_s becomes eq. 2. (Supplementary Fig. 11a) t_{res} can be obtained by fitting the curve against $v(t)$ in unsteady-state permeation but not in steady-state permeation.

$t = 0$ and t_{end} are the arrival and departure time of the α'/α interface at the edges of the capillary attached to the crystal surface. The permeation area (S) increasing with time is calculated by dividing the area into m number of rectangular regions (m=1000). The permeated gas volume ($V(t)$) is an integration of $v_i(t)$. (Supplementary Equations 3 and 4) (Supplementary Figs. 11b and 11c) It is noted that accumulation of the permeated volume ($v_i(t)$) was delayed by $t_{i\text{-}1}$ ($(i\text{-}1)/m \times t_{end}$). The position of the interface (x_i) at t_i ($i/m \times t_{end}$) was determined by making videos under a microscope; the initial time of observing the oil transfer is denoted as t_{mov}.

Silicone oil in a capillary was allowed to move after the passage of a waiting time (t_w) because additive pressure (Δp) is needed to exceed the viscous resistance from the inner wall. (Supplementary Equations 6 and 7, Supplementary Fig. 12 and Supplementary Tables 6,7)

t_{res} resulted in -10 – 6 msec and as -30 – 10 msec from curve fittings against the permeation amount in Figs. 4d and 4e, respectively, by using eqs. 4 and 5. (Supplementary Figs. 13-16 and Supplementary Tables 8, 9) Therefore, none of t_{res} plugs into the immediate gas permeation without delay after the crystal phase transition.

Supplementary References:

[1] Takamizawa, S., Takasaki, Y. & Miyake, R. *J. Am. Chem. Soc*. **132** (9), 2862-2863 (2010).
[2] Crowe, C. T., Elger, D. F., Roberson, J. A. *Engineering Fluid Mechanics 8th Ed.* John Wiley&Sons Inc. New York, 2005, page 379.
